# Chitosan-Tripolyphosphate Nanoparticles Prepared by Ionic Gelation Improve the Antioxidant Activities of Astaxanthin in the In Vitro and In Vivo Model

**DOI:** 10.3390/antiox11030479

**Published:** 2022-02-28

**Authors:** Eun Suh Kim, Youjin Baek, Hyun-Jae Yoo, Ji-Soo Lee, Hyeon Gyu Lee

**Affiliations:** 1 Department of Food and Nutrition, Hanyang University, Seoul 04763, Korea; copadiet@gmail.com (E.S.K.); jyyj161126@hanyang.ac.kr (Y.B.); hyunjaey@naver.com (H.-J.Y.); 2 Korean Living Science Research Center, Hanyang University, Seoul 04763, Korea

**Keywords:** astaxanthin, nanoencapsulation, chitosan nanoparticle, stability, antioxidant activity, bioavailability

## Abstract

The present study aimed to investigate the effects of chitosan (CS)-tripolyphosphate (TPP) nanoparticles (NPs) on the stability, antioxidant activity, and bioavailability of astaxanthin (ASX). ASX-loaded CS-TPP NPs (ACT-NPs) prepared by ionic gelation between CS (0.571 mg/mL) and TPP (0.571 mg/mL) showed 505.2 ± 184.8 nm, 20.4 ± 1.2 mV, 0.348 ± 0.044, and 63.9 ± 3.0% of particle size, zeta potential, polydispersity index and encapsulation efficiency, respectively. An in vitro release study confirmed that the release of ASX in simulated gastric (pH 1.2) and intestinal (pH 6.8) fluid was prolonged within ACT-NPs. The in vitro antioxidant activities of ACT-NPs were significantly improved compared with free ASX (FA) (*p* < 0.05). Furthermore, the cellular and in vivo antioxidant analysis verified that ACT-NPs could enhance the cytoprotective effects on the BHK-21 cell line and demonstrate sustained release properties, leading to prolonged residence time in the rat plasma. The results suggest that the stability, antioxidant properties, and bioavailability of ASX can be effectively enhanced through encapsulation within CS-TPP NPs.

## 1. Introduction

**Figure 1:**
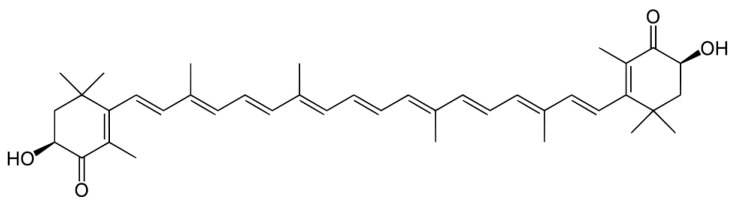
Structure of astaxanthin.

Astaxanthin (3,3′-dihydroxy-β-β′-carotene-4-4′-dione, ASX), a natural carotenoid pigment generally abundant in marine organisms including shrimp, crab, shuck, and salmon, is an efficient natural antioxidant [1]. The polar structure of ASX, due to the hydroxyl (OH) and the carbonyl (C=O) groups of each ionic ring, has been reported to show higher antioxidative activities compared with β-carotene, tocopherol, and ascorbic acid (Figure 1) [2,3]. Moreover, various physiological activities of ASX such as anti-inflammatory, anticancer, and immunological activity, expressed from the high antioxidant effect, have been reported [4]. However, in spite of the various beneficial effects, poor aqueous solubility, which results in a low in vivo absorption rate, has been reported as a constraint to ASX’s application as a potential functional material in food [5]. Moreover, a previous study reported that ASX decomposes easily with heat, light, and oxygen due to its eleven conjugated carbon–carbon double bonds [6]. Thus, overcoming the insolubility and instability of ASX has been studied as an essential task to be solved in order for it to be used as an efficient food or pharmaceutical ingredient [7,8].

Nanoencapsulation is the technology of entrapping one bioactive compound within a nanosized structure made of the other wall materials [9]. Because of the protective effects of the wall materials and the increased surface areas arising from nanosized carriers, nanoencapsulation has been widely used for potential delivery systems to enhance the solubility, stability, and absorption of bioactive materials [10]. Recently, several studies applying various types of nanoencapsulation techniques to ASX have been reported, such as poly lactic-co-glycolic acid (PLGA) nanoparticles (NPs) [11] and lipid-based nanocarriers including nanoliposomes [12], nanoemulsions [13], and nanostructured lipid carriers [14]. While these studies showed the results in enhancing the stability, antioxidant activities, and absorption of ASX, the process and materials were regarded as inappropriate for food application, requiring a high degree of safety owing to the use of synthetic polymers and surfactants [15,16]. Moreover, liposomes have several limitations such as poor stability and low encapsulation efficiency [17].

On the other hand, NPs using natural polysaccharides such as chitosan (CS) are regarded as a suitable technique for food applications because of their nontoxic, biodegradable, biocompatible, and economical manufacturing process [18]. CS, generated by the deacetylation of chitin obtained from crab or shrimp shells, is a cationic, biodegradable biopolymer. Due to its cationic charge properties in acidic solutions, CS can produce NPs through electrostatic interaction with oppositely charged anionic polymers. CS-NPs have been studied for encapsulating various functional materials such as resveratrol, quercetin, and curcumin to improve their water solubility and stability against heat and gastrointestinal environments [19,20,21]. In addition, CS-NPs have been extensively investigated as a delivery system due to their positively charged properties, which have a powerful affinity for anionic cell membranes, leading to enhanced mucoadhesive potential and bioavailability, both ex vivo and in vivo [22]. However, only a few studies have attempted to study the encapsulation of ASX within CS-NPs to enhance its stability, antioxidant activity, and bioavailability [23,24].

Therefore, the aim of this study was to encapsulate ASX within CS-NPs to investigate the influence of CS-NPs on the biodelivery and antioxidative activities of ASX. ASX-loaded CS-NPs were prepared using sodium tripolyphosphate (TPP) which is one of the most effective nontoxic anionic polymers for ionic crosslinking with cationic amino groups of CS [21]. The physicochemical features, including those of ASX-loaded CS-TPP NPs (ACT-NPs) were investigated, including particle size, zeta potential (ZP), polydispersity index (PDI), encapsulation efficiency (EE), and in vitro release characteristics. Furthermore, the impact of ACT-NPs on the antioxidant activities of ASX were investigated by lipid peroxidation using ferric thiocyanate (FTC) and thiobarbituric acid (TBA) methods, 1,1-diphenyl-2-picrylhydrazyl (DPPH) radical scavenging assay, cytoprotective properties, and in vivo ferric reducing ability of plasma (FRAP) assay.

## 2. Materials and Methods

### 2.1. Materials

Chitosan (CS, 50–190 kDa, 24 cps, 95% deacetylated), sodium triphosphate (TPP), 2,2-Diphenyl-1-pikryl-hydrazyl (DPPH), linoleic acid, and sodium dodecyl sulfate (SDS) were purchased from Sigma-Aldrich Co. (St. Louis, MO, USA). Astaxanthin (ASX) was obtained from Neo Cremar Co. (Sungnam, Korea). Baby hamster kidney (BHK)-21 cells were obtained from the Korean Cell Line Bank (Seoul, Korea). Dulbecco’s Modified Eagle’s culture medium (DMEM) and fetal bovine serum were obtained from Gibco Invitrogen Co. (Grand Island, NY, USA). Penicillin-streptomycin and phosphate-buffer saline (PBS) were obtained from Lonza (Walkersville, MD, USA). 3-(4,5-dimethylthiazol-2-yl)-2,5-diphenyltetrazolium bromide (MTT) was purchased from Sigma-Aldrich Co. (St. Louis, MO, USA). All other chemicals were of reagent grade, and all solvents were of HPLC grade.

### 2.2. Preparation of ACT-NPs

ACT-NPs were formulated by ionic gelation with some minor improvements from a previous study [25]. CS, ASX, and TPP were suspended in 1% (*v*/*v*) acetic acid, ethanol, and deionized water (DW), respectively, at the final concentration (Table 1). Then, 0.5 mL of ASX solution was combined with 2 mL of CS solution under magnetic stirring at 1000 rpm for five min, and then 4.5 mL of TPP solution was added using a master flex pump (Master flex 77200-60, Cole Parmer Inc., Vernon Hills, IL, USA) at 1 mL/min flow rate. 

### 2.3. Characterization of ACT-NPs

#### 2.3.1. Particle Size, ZP, and PDI of ACT-NPs

Particle size, ZP, and PDI of NPs were analyzed by dynamic light scattering (DLS) using Malvern Zetasizer Nano ZS (Malvern Instruments Ltd., Malvern, Worcestershire, UK). ACT-NPs suspension obtained immediately after fabrication were transferred into DTS 1060C disposable zeta cell for the measurements. All measurements were carried out in multiple narrow modes at 25 ± 1 °C. 

#### 2.3.2. Encapsulation Efficiency of ACT-NPs

ACT-NPs suspension was ultracentrifuged for 30 min at 30,000× *g* and 4 °C (Optima TL, Beckman Instruments, Fullerton, CA, USA). After separating the supernatant, ACT-NPs were collected and lyophilized (FD8508; Ilshin Co., Seoul, Korea). Freeze-dried ACT-NPs (14 mg) were suspended in 2.5 mL of acetone to extract ASX, followed by UV measurement at 478 nm (Biomate 3S; Thermo Scientific, Madison, WI, USA). The EE of ACT-NPs was calculated using the following equation:

(1) $$\mathrm{EE}\;\left(\%\right)=\frac{\mathrm{Actual}\;\mathrm{amount}\;\mathrm{of}\;\mathrm{ASX}\;\mathrm{encapsulated}\;\mathrm{in}\;\mathrm{ACT}-\mathrm{NPs}}{\mathrm{Theoretical}\;\mathrm{amount}\;\mathrm{of}\;\mathrm{ASX}\;\mathrm{encapsulated}\;\mathrm{in}\;\mathrm{ACT}-\mathrm{NPs}}\times 100$$

### 2.4. In Vitro Release Properties

Simulated gastric fluids (SGF, pH 1.5) containing 0.1 M hydrogen chloride (HCl) and 0.05 M sodium chloride and simulated intestinal fluids (SIF, pH 6.8) containing 0.1 M sodium hydroxide and 0.05 M sodium dihydrogen phosphate buffer were utilized to study ASX release from ACT-NPs. Briefly, freeze-dried ACT-NPs (20 mg) were suspended in 5 mL of SGF and SIF, respectively, and kept in a horizontal incubator at 37 °C, under stirring at 100 rpm. To analyze the release rate at the predetermined time, 2 mL of each sample was withdrawn at 2, 6, and 12 h, and ASX was extracted using chloroform. The concentration of released ASX was measured by a UV spectrophotometer as aforementioned. The ASX release ratio was calculated using the following equation:

(2) $$\mathrm{Release}\;\mathrm{rate}\;\left(\%\right)=\frac{\mathrm{Amount}\;\mathrm{of}\;\mathrm{ASX}\;\mathrm{relesed}\;\mathrm{from}\;\mathrm{ACT}-\mathrm{NPs}}{\mathrm{Amount}\;\mathrm{of}\;\mathrm{ASX}\;\mathrm{initally}\;\mathrm{encapsulated}\;\mathrm{in}\;\mathrm{ACT}-\mathrm{NPs}}\times 100$$

### 2.5. In Vitro Antioxidant Activity

#### 2.5.1. Lipid Peroxidation Assays

A linoleic acid model system was utilized to assess the antioxidative activities of non-encapsulated and encapsulated ASX [26]. Measures of 0.143 g of tween 20, 10 mL of potassium phosphate buffer (20 mM, pH 7.0), 0.08 mL of linoleic acid, and 10 mL of 30% ethanol were used to produce the model system for lipid peroxidation inhibitory activity. The volume was adjusted to 25 mL with DW. Accurately weighed amounts of the free ASX (FA) and ACT-NPs containing 2 mg of ASX were added to the linoleic acid model system as the test samples. The linoleic acid model system without FA and ACT-NPs was used as a control. All samples were incubated at 40 ± 1 °C in a dark room to accelerate oxidation and then withdrawn at regular time intervals (2, 4, 6 days). To estimate the degree of oxidation, the FTC and TBA values were determined.

The FTC method was carried out in accordance with the previous study with minor modification [27]. In the FTC method, the quantity of peroxide produced at the initial stage of the peroxidation of linoleic acid is estimated. Fe^2+^ is oxidized to Fe^3+^ by the peroxides, and a thiocyanate complex is produced when Fe^3+^ reacts with thiocyanate [28]. The above linoleic reaction solution (20 μL) withdrawn at regular time intervals was mixed with 20 μL of 20 mM ferrous chloride solution, 20 μL of 30% (*w*/*v*) ammonium thiocyanate in 3.5% (*v*/*v*) HCl, and 1.14 mL of 75% (*v*/*v*) ethanol. After incubation for three min at 40 °C, the absorbance of the mixture was analyzed at 500 nm using ELISA microplate reader (ELx800UV, Bio-Tek Instrument Inc., Windoski, VT, USA). The increased absorbance demonstrates increased lipid peroxidation. 

The TBA value was measured using a method described by Ohkawa (1979) [29]. TBA value reflects the extent of lipid peroxidation associated with the formation of malonaldehyde, an essential biomarker to evaluate the secondary stage of linoleic acid peroxidation [30]. Briefly, the samples (200 μL) withdrawn at predetermined time intervals were added to 0.2 mL of 8.1% SDS, 1.5 mL of 20% (*v*/*v*) acetic acid adjusted to pH 3.5, and 1.5 mL of 0.8% (*w*/*v*) TBA. After these mixtures were adjusted to 4 mL with DW, the mixture was reacted for one h at 4 °C before being heated in the dark for one h at 95 °C. Finally, the absorbance of the mixtures was analyzed at 532 nm by ELISA microplate reader after cooling at room temperature. The inhibition activity toward lipid peroxidation performed using FTC and TBA methods was calculated by the following equation:

(3) $$\mathrm{Antioxidant}\;\mathrm{activity}\;\left(\%\right)=\frac{\mathrm{Ac}-\mathrm{As}}{\mathrm{Ac}}\times 100$$

where Ac is the absorbance of the control and As is the absorbance of the sample.

#### 2.5.2. DPPH Radical Scavenging Activity

DPPH radical scavenging capacity was measured according to a previous study with some adjustments [31]. FA and ACT-NPs during storage, they were incubated at 25 °C for 30 days and then withdrawn at predetermined time intervals to measure their antioxidant activity. A measure of 0.7 mL of DPPH ethanol solution (0.1 mM) was mixed with 0.3 mL of samples, followed by the incubation in a shaking water bath at 37 °C for one h. The absorbance was measured at 517 nm immediately after centrifugation (Combi 408, Hanil Co., Seoul, Korea) at 10,000 rpm for 10 min. The DPPH radical scavenging activity was calculated according to the equation:

(4) $$\mathrm{DPPH}\;\mathrm{radical}\;\mathrm{scavenging}\;\mathrm{activity}\;\left(\%\right)=\frac{\left(C-CB\right)-\left(S-SB\right)}{C-CB}\times 100$$

where *C* is the absorbance of the control, *CB* the absorbance of the control blank, *S* the absorbance of the sample, and *SB* the absorbance of the sample blank.

### 2.6. Ex Vivo Antioxidant Activity of ACT-NPs

The MTT test was conducted to analyze cell viability in order to determine whether the ACT-NPs had any protective and cytotoxic effects [32]. A measure of 180 μL of the baby hamster kidney fibroblast cell line BHK-21 were seeded in 96-well plate at a density of 1.0 × 10^4^ cells/well and cultured at 37 °C under a humidified atmosphere with 5% CO_2_. After BHK-21 cells had reached 80% confluence, cells were treated with 20 μL of FA, blank NPs (BNPs), and ACT-NPs suspensions at concentrations ranging from 12.5 to 500.0 μg/mL, followed by an incubation for 24 h. The cells were washed with PBS, after discarding the cell culture medium and 1 mM hydrogen peroxide (H_2_O_2_) was applied to generate oxidative stress in the cells for one hour. 

Then, 20 μL of freshly prepared MTT reagent (5 mg/mL) was introduced to each well and reacted at 37 °C for another 4 h to simulate the formation of purple formazan crystals created by the transformation of yellow tetrazolium bromide due to mitochondrial succinate dehydrogenase in live cells. The supernatant was discarded after centrifugation for five min at 400× *g*, and dimethyl sulfoxide was added to completely dissolve formazan. The UV absorbance of formazan were determined at 540 nm using ELISA microplate reader to assess cell viability.

### 2.7. In Vivo FRAP Assay

Sprague−Dawley (SD) rats (male, 6 weeks old) were obtained from Woojung Bio Co., Ltd. (Suwon, Korea). The rats were housed with a 12 h light–dark cycle at a humidity of 55 ± 5% and temperature of 22 ± 2 °C. They were acclimatized with free access to standard chow and tap water for 1 week. Hanyang University’s IACUC guidelines were followed for all animal experiments. Before the study, all rats were starved overnight, and then they were randomly separated into three groups of six rats each. One group was treated with freshly prepared ACT-NPs at a dose of 10 mL/kg body weight (BW). Another group was given BNPs, which were prepared without ASX. The last group was treated with FA. At 2, 4, 6, 8, 10, and 12 h after sample treatment, aliquots of blood from retro-orbital puncture were collected in 1 mL EDTA tubes, followed by the centrifugation for 10 min at 2000× *g*. Until analysis, the plasma samples were promptly frozen at −70 °C. 

The antioxidant properties in the plasma samples were evaluated using FRAP assay, which was modified slightly from Benzie and Strain (1996) [33]. FRAP solution, containing acetate buffer (pH 3.6, 300 mM), 2,4,6-tripyridyl-s-triazine (10 mM) in HCl (40 mM), and FeCl_3_·6H_2_O (20 mM) at a ratio of 10:1:1 (*v*/*v*/*v*), was freshly produced and warmed at 37 °C prior to the assay. Plasma samples (30 μL) were reacted with a mixture of FRAP reagent (900 μL) and DW (90 μL) in the dark for 30 min at 37 °C. The antioxidant activity in the plasma samples was analyzed by reading UV absorption of the mixture at 595 nm using a Synergy HT multi microplate reader.

### 2.8. Statistical Analysis

All experiments were conducted in triplicate, and all data are expressed as the mean ± standard deviation (SD). To assess the significant differences across all groups, one-way ANOVA followed by Duncan’s multiple range test (SPSS Version 21.0, SPSS Inc., Chicago, IL, USA) was used. Significant differences in DPPH radical scavenging effect between two groups were determined using a Student’s *t*-test. *p*-values below 0.05 were considered statistically significant.

## 3. Results and Discussion

### 3.1. Characteristics of ACT-NPs

#### 3.1.1. Particle Size and ZP of ACT-NPs

The ACT-NPs were formulated by various ratios of the cationic CS and anionic TPP with the identical concentration of ASX as shown in Table 1. In this study, TPP higher than 0.571 mg/mL and lower than 0.429 mg/mL were considered unsuitable for the ACT-NPs preparation due to their irregular NPs formation as predetermined in our preliminary experiments. At the identical CS concentration of 0.571 mg/mL, the particle size of ACT-NPs ranged from 483.9 ± 148.4 to 505.2 ± 184.8 nm with TPP concentration ranging from 0.571 to 0.468 mg/mL, followed by a significant increase to 653.8 ± 215.1 nm with the decrease of TPP concentration to 0.429 mg/mL (*p* < 0.05). While NPs are generally defined as particles with a diameter of 100 nm or less, the particle size range in the food industry has been expanded to 1000 nm as acceptable food-grade wall materials are likely to perturb the uniformity and small size of NPs due to their comparably low purity and large molecular weight [34]. According to a previous study, the physicochemical properties of NPs, formed by ionic gelation between positively and negatively charged polymers, may be affected by the relative proportions of charged groups [35]. As a result, TPP concentrations above or below the appropriate level at consistent CS concentrations would interrupt the charge balance between CS and TPP and influence the physicochemical characteristics of NPs, such as particle size, ZP, and propensities of particle aggregation. A similar result has been reported that a decrease in the mass ratio of CS and TPP from 5:1 to 2:1 led to an increase in particle size of CS-NPs [36]. This can be explained by the relatively increased amount of TPP, therefore, the superfluous TPP in suspension can produce larger NPs [37]. However, a decrease in TPP concentration to 0.429 mg/mL demonstrated a significant increase in particle size (*p* < 0.05). These results highlight that particle size of CS-TPP NPs arguably varies under different compositions such as concentration and ratio of CS and TPP.

The ZP values of ACT-NPs significantly increased from 20.4 ± 1.2 to 30.6 ± 0.6 mV with a decrease of TPP concentration (*p* < 0.05). The ZP values of NPs depend on the composition of charged groups as aforementioned. At a higher TPP concentration, the neutralization degree of the protonated amino groups is increased, leading to lower ZP values of CS-TPP NPs [38]. Moreover, as CS and TPP carry positive and negative charges, respectively, it is reasonable to conclude that an increase in TPP concentration led to a decrease in the positive charge of NPs. As ZP illustrates the surface charge of NPs, better dispersion stability is associated with a higher ZP value due to the electric repulsiveness between each NP [18]. Furthermore, it has been shown that NPs with ZP values of either more than +20 mV or less than −20 mV are found to be stable [39]. The dispersion stability of NPs was confirmed to be around 0.3 of PDI level, which is the recommended level for the delivery system [40]. Therefore, all ACT-NPs formulations demonstrated in the Table 1 can be considered stable NPs. 

#### 3.1.2. Encapsulation Efficiency of ACT-NPs

The EE of ACT-NPs significantly increased with an increase in TPP concentration as shown in Table 1 (*p* < 0.05). This could be due to the number of crosslinking units at different TPP concentrations. An increase in TPP concentration up to a certain ratio is associated with better crosslinking ability between CS and TPP which causes a strong affinity [38]. A strong affinity between CS and TPP would lead to ASX being tightly bound with CS-TPP structure, therefore ASX became difficult to release rapidly from the NPs. On the other hand, with a further increment in the TPP concentration, EE could be decreased due to a more compact structure between CS and TPP which could hinder the interaction between TPP and the core material for CS binding [38]. However, as the ACT-NPs presented in this study were prepared with the optimal ratio of CS and TPP for NP formation, therefore, the ACT-NPs with the highest concentration of TPP (0.571 mg/mL) showed significantly higher EE (63.9%) (*p* < 0.05). According to the above discussion, a CS and TPP ratio of 1:1 was chosen for further study as the ACT-NPs prepared at this ratio resulted in the highest EE with acceptable particle size and distribution. 

### 3.2. In Vitro Release Properties

**Figure 2:**
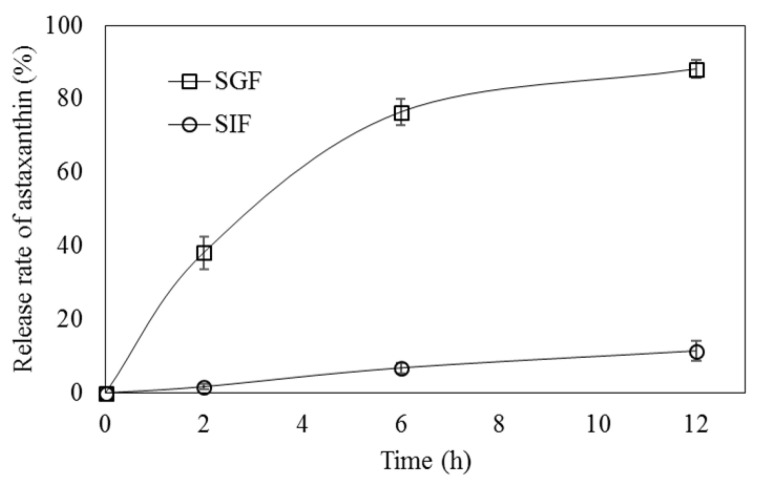
Release rate of astaxanthin from CS-TPP NPs in simulated gastric (SGF, pH 1.2) and intestinal (SIF, pH 6.8) fluid.

In the SGF environment, the release rate of ASX from ACT-NPs gradually increased without burst release during the incubation period, and the release rate reached 88.1 ± 2.4% after 12 h (Figure 2). The release of the core material was primarily affected by the degradation of NP structure. The crosslinking between CS and TPP can be affected as the charge state of the wall materials can be changed by the external pH environment where NPs are dispersed [20]. Moreover, NPs with high ZP values tend to have strongly charged ions on the surface which indicate a higher resistance to the degradation of NPs in the presence of an acidic environment [41]. In other words, NPs with low ZP value are susceptible to degradation in an acidic environment which leads to a higher release of the core material. As aforementioned, the ZP value of ACT-NPs prepared with a 1:1 ratio of CS and TPP was 20.4 ± 1.2 mV, which is lower than other formulations. Thus, this denotes that a high ASX release of ACT-NPs in SGF could be explained by their reduced electrostatic attraction between CS and TPP in an acidic environment. Another possible explanation for the increased ASX release in SGF is the solubility of CS in an acidic environment. In general, as CS is soluble in the acidic pH range, the interaction within CS-TPP NPs prepared by ionic gelation tends to reduce in SGF and accelerate the release of ASX [42].

On the other hand, the release of ASX from ACT-NPs was not induced in the SIF environment, and the release rate reached 11.4 ± 2.7% after 12 h (Figure 2). This difference can be explained by two main interaction mechanisms between CS-NPs and the SIF environment. One was deprotonation of CS molecules at neutral pH, which could stabilize the NP structure due to the formation of more hydrogen bonds [43]. Another possibility was the insolubility of CS at neutral pH. The structure of ACT-NPs might be sustained without CS dissolution since CS solubility in neutral conditions is substantially lower than in acidic environments. Likewise, the previous study observed the behavior of CS-NPs in different pH media (pH 1.2, 6.5, 7.2) to investigate the release profile of insulin. They affirmed that the CS-NPs showed a lower release of insulin at pH 6.5 than pH 1.2, due to the solubility of chitosan in different media [42]. Therefore, ACT-NPs prepared using CS and TPP appeared to have prolonged degradation in SIF, as a result, leading to a sustained ASX release.

### 3.3. In Vitro Antioxidant Activity

**Figure 3:**
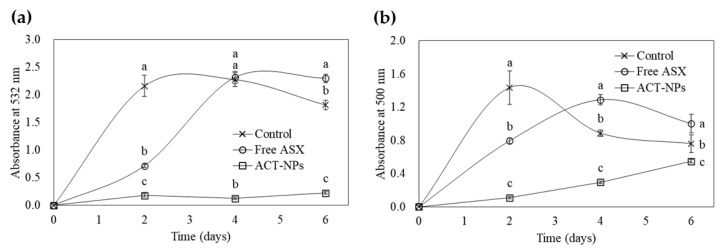
Antioxidant activity of free ASX and ACT-NPs in the linoleic acid peroxidation system using ferric thiocyanate method (**a**) and thiobarbituric acid (**b**) method. ^a–c^ Means with different letters are significantly different (*p* < 0.05).

The results of lipid peroxidation analysis using FTC and TBA methods are shown in Figure 3a,b. The absorbance of the control started to increase rapidly within 2 days. However, it gradually decreased when it reached the maximum level. The absorption values of the FA constantly increased and reached a maximum level within 4 days, but slightly decreased over time. However, the absorption values of ACT-NPs increased slightly over 6 days and showed significantly lower levels than others within the experimental period (*p* < 0.05). TBA methods showed similar results. While the absorbances of control and FA increased rapidly, reaching the maximum level at 2 and 4 days, respectively, and decreased gradually over time. On the other hand, the absorbance of the ACT-NPs maintained significantly lower levels without gradual increase during the experimental period (*p* < 0.05).

**Figure 4:**
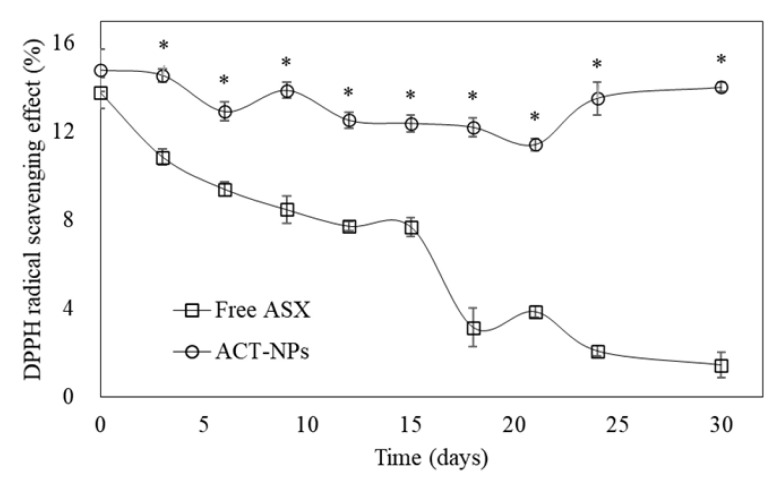
Antioxidant activity of free ASX and ACT-NPs using DPPH radical scavenging method. Single asterisk indicates significant differences (*p* < 0.05) between two groups at each time point using the Student’s *t*-test.

As shown in Figure 4, the DPPH radical scavenging effect of both FA and ACT-NPs was 13.77 and 14.80% on day 0, respectively, which showed no significant differences. However, the effect of FA significantly decreased to 0.21% on day 30, whereas ACT-NPs showed constant DPPH radical scavenging effect during the incubation time (*p* < 0.05).

The results obtained from lipid peroxidation and DPPH assay indicate that the ACT-NPs maintained antioxidant activity of ASX effectively, compared with FA. Moreover, ACT-NPs showed strong inhibitory effects on linoleic acid peroxidation of 92% within 2 days both in FTC and TBA methods, indicating that ACT-NPs were effective in hindering both initial and secondary stage oxidation of linoleic acid. Prolonged antioxidant activity of ACT-NPs can be explained by three different mechanisms: increased solubility, sustained release property, and large surface area. The solubility of the core material is one of the key factors affecting the antioxidant activity as it reacts with free radicals in a dissolved state [44]. For example, a previous study confirmed that the antioxidant activity of kaempferol was dramatically increased due to encapsulation within CS-NPs as its water solubility was increased [45]. In addition, CS-NPs with a prolonged release property could enhance the antioxidant activity by maintaining the stability of the core material [46]. Furthermore, the enhanced antioxidant activity of ACT-NPs is likely associated with the large surface area of CS-TPP NPs that allows ASX molecules to interact with the reaction medium more effectively [47]. Thus, this study confirmed that the antioxidant activity of ASX was improved by encapsulating within CS-TPP NPs.

### 3.4. Ex Vivo Antioxidant Activity of ACT-NPs

**Figure 5:**
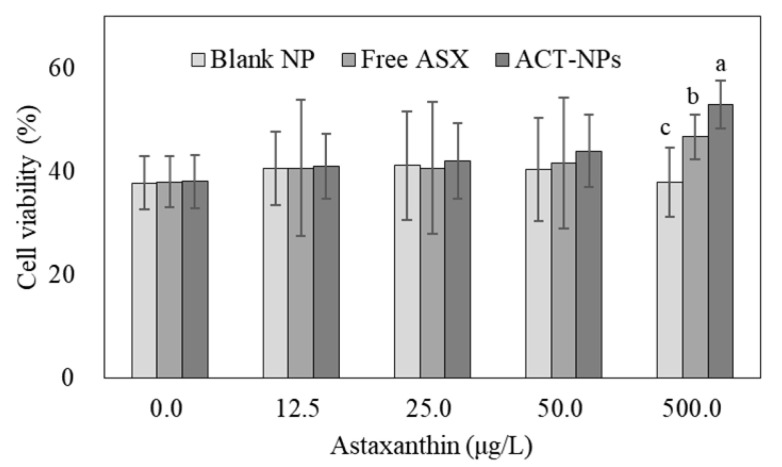
Cytoprotective effect of blank NPs, free ASX, and ACT-NPs after hydrogen peroxide-induced damage to BHK-21 cell line. ^a–c^ Means with different letters are significantly different (*p* < 0.05).

The viability of BHK-21 cells was less than 40% due to the H_2_O_2_-induced damage and the viability was not improved with a treatment of less than 50 μg/L of ASX or ACT-NPs containing the same amount of ASX, as shown in Figure 5. However, the cell viability was significantly increased at 500 μg/L of FA (46.7 ± 4.3%) (*p* < 0.05), and the activity of ASX was markedly increased by encapsulation within ACT-NPs (52.9 ± 4.7%). Apparently, ACT-NPs at 500 μg/L of ASX were effective for the protection of BHK-21 cells against H_2_O_2_-induced injury. 

Since the antioxidant activity of ASX is activated after cellular uptake, these results can be clarified by the cell permeation characteristics of CS-NPs. The inside of the cell membrane is negatively charged due to the Na+-K+ pump which consumes energy from hydrolysis to pump Na+ and K+ across their electrochemical gradients [48]. As CS carries a positive charge because it has amino groups in its structure, the electrostatic interaction between cationic CS and anionic cell surfaces might be one of the key factors that influence the cell permeability properties of CS-NPs [49]. Thus, prolonged contact between the ACT-NPs and cell surfaces enhances the absorption capacity of the CS-NPs, and therefore increases the antioxidant activity of ASX.

### 3.5. In Vivo FRAP Assay

**Figure 6:**
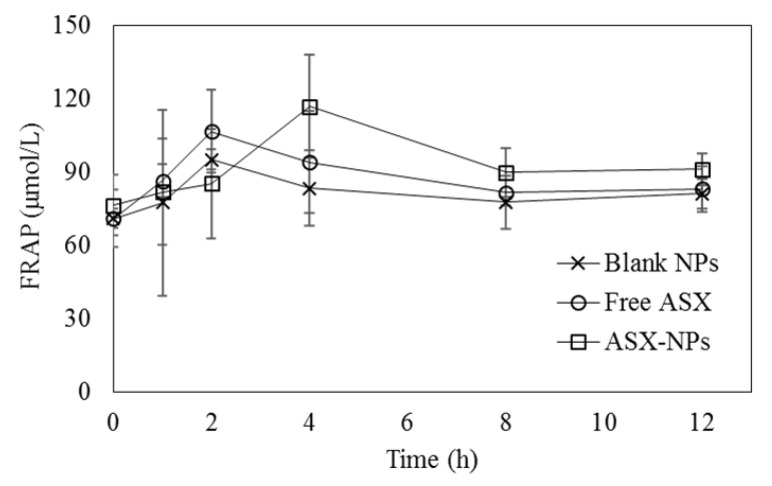
Changes in FRAP values in rat plasma after a single dose of blank NPs, free ASX, and ACT-NPs.

The change in the FRAP value in SD rats after oral administration of BNPs, ASX, and ACT-NPs is illustrated in Figure 6. The FRAP values of BNP- and FA-treated rats increased quickly within two h to their highest levels, 95.26 ± 4.00 and 106.68 ± 17.93 μmol/L, respectively, while the FRAP value of ACT-NP-fed rats only increased to 85.33 ± 22.45 μmol/L within the same time period. However, the FRAP values were inverted after 2 h of oral administration. Briefly, the FRAP values of ASX-fed rats decreased to 94.07 ± 20.85 μmol/L at 4 h, continuously decreased over time and returned to their starting value after 8 h, demonstrating that ASX has low stability in an alkaline condition [50]. The FRAP value of the ACT-NPs, on the other hand, constantly increased and exceeded the FRAP value of FA after 4 h. Although the FRAP value of ACT-NPs started to decrease 4 h after administration, the antioxidant activity of ACT-NPs was consistently superior to that of FA. 

A previous study demonstrated that the bioavailability of orally fed ASX was merely 12% of ASX infused by intraperitoneal injection, indicating that ASX has low oral bioavailability due to its lipophilic characteristic [51]. This limitation of ASX can be overcome through encapsulation within CS-TPP NPs. This is likely due to the sustained release of ASX from CS-TPP NPs, as mentioned in the in vitro release studies, indicating that CS-TPP NPs are effective in protecting ASX from degradation during the absorption process in the gastrointestinal tract. Moreover, as encapsulation within CS-NPs has been reported as an effective way to improve the solubility of the core material, ACT-NPs can enhance the solubility of ASX, thus promoting its oral bioavailability [45]. Therefore, encapsulation within CS-TPP NPs may have the potential to be used as an oral delivery system for ASX to maintain its antioxidant power after oral administration.

## 4. Conclusions

In the present study, we encapsulated ASX within ACT-NPs using CS and TPP by ionic gelation method. The ACT-NPs showed acceptable physicochemical characteristics such as particle size, ZP, PDI, and EE. The physicochemical characteristics have shown that the interaction between CS and TPP for NP formation is markedly influenced by the ratio between CS and TPP. CS-TPP NPs with a 1:1 ratio of CS and TPP were found to enhance the release properties, in vitro and in vivo antioxidant activities, and the cytoprotective effect of ASX. ACT-NPs showed sustained release of ASX (11.4 ± 2.7%) in the SIF environment, indicating that CS-TPP NPs have the potential to increase the stability of ASX. This tendency was consistent with the results of antioxidant activities in vitro, ex vivo, and in vivo, showing that CS-TPP NPs can successfully influence the antioxidant activities and oral bioavailability of ASX compared with FA. The results suggest that CS-TPP NPs with desirable NPs characteristics may be used as a potential oral delivery system aimed at improving the stability, antioxidant activities, and bioavailability of ASX.

## Figures and Tables

**Table 1:** Preparation conditions and characteristics of astaxanthin-loaded chitosan nanoparticles prepared with different concentrations of TPP. ^a–d^ Different letters in the same column indicate significant differences (*p* < 0.05).

CS:TPP Ratio	Chitosan (mg/mL)	TPP (mg/mL)	ASX (mg/mL)	Particle Size (nm)	Zeta Potential (mV)	Polydispersity index	Encapsulation Efficiency (%)
1:1	0.571	0.571	0.036	505.2 ± 184.8 ^b^	20.4 ± 1.2 ^d^	0.348 ± 0.044	63.9 ± 3.0 ^a^
1.1:1	0.571	0.514	0.036	486.0 ± 98.9 ^b^	22.6 ± 0.6 ^c^	0.334 ± 0.042	46.6 ± 6.1 ^b^
1.2:1	0.571	0.468	0.036	483.9 ± 148.4 ^b^	28.5 ± 0.8 ^b^	0.322 ± 0.005	34.5 ± 12.8 ^c^
1.3:1	0.571	0.429	0.036	653.8 ± 215.1 ^a^	30.6 ± 0.6 ^a^	0.335 ± 0.027	18.6 ± 4.8 ^d^

## Data Availability

The data presented in this study are available within the article.
